# The first international conference on *SYNGAP1*-related brain disorders: a stakeholder meeting of families, researchers, clinicians, and regulators

**DOI:** 10.1186/s11689-018-9225-1

**Published:** 2018-02-05

**Authors:** Monica Weldon, Murat Kilinc, J. Lloyd Holder, Gavin Rumbaugh

**Affiliations:** 1 Bridge-the-GAP—SYNGAP Education and Research Foundation (ERF), Cypress, TX USA; 2 Graduate School of Chemical and Biological Sciences, The Scripps Research Institute, Jupiter, FL USA; 3 Jan and Dan Duncan Neurological Research Institute and Department of Pediatrics, Division of Neurology and Developmental Neuroscience, Baylor College of Medicine, 1250 Moursund St. Suite 1150, Houston, TX 77030 USA; 4 Department of Neuroscience, The Scripps Research Institute, 130 Scripps Way, #3B3, Jupiter, FL 33458 USA

**Keywords:** Rare disorder, Stakeholder meeting, *SYNGAP1*, Neurodevelopmental disorders, Epilepsy, Intellectual disability, Autism spectrum disorder

## Abstract

**Background:**

Pathologic mutations in *SYNGAP1* cause a genetically defined form of intellectual disability (ID) with comorbid epilepsy and autistic features. While only recently discovered, pathogenicity of this gene is a relatively frequent genetic cause of classically undefined developmental delay that progresses to ID with commonly occurring comorbidities.

**Main body:**

A meeting of 150 people was held that included affected individuals and their caregivers, clinicians that treat this and related brain disorders, neuroscientists that study *SYNGAP1* biology or the function of related genes, and representatives from government agencies that fund science and approve new medical treatments. The meeting focused on developing a consensus among all stakeholders as to how best to achieve a more fundamental and profound understanding of *SYNGAP1* biology and its role in human disease.

**Short conclusion:**

From all of these proceedings, several areas of consensus emerged. The clinicians and geneticists agreed that the prevalence of epilepsy and sensory processing impairments in *SYNGAP1*-related brain disorders approached 100%. The neurobiologists agreed that more basic research is needed to better understand the molecular and cellular functions of the *Syngap1* gene, which will lead to targets for therapeutic intervention. Finally, everyone agreed that there is a pressing need to form a robust patient registry as an initial step toward a prospective natural history study of patients with pathogenic *SYNGAP1* variants.

## Background

### Clinical background

Heterozygous loss-of-function variants (i.e., nonsense mutation, large deletion, frameshift) in *SYNGAP1* cause a genetically defined form of intellectual disability (ID) termed *autosomal mental retardation type 5* (MRD5; phenotype MIM 612621; gene/locus MIM 603384). Common phenotypes in MRD5 include cognitive impairment, severely impaired expressive and receptive language, behavioral deficits, and epilepsy [1–3]. MRD5 individuals are also frequently diagnosed with autism spectrum disorder (ASD) and attention deficit hyperactivity disorder. *SYNGAP1* loss-of-function variants are surprisingly common, with the incidence reported as 1–4/10,000 individuals, or approximately 0.5–1.0% of all ID cases, making it one of the most common causes of ID with epilepsy [1, 4, 5]. There are currently ~ 200 known patients, which are discovered through genetic sequencing, though estimates of incidence suggest that there are thousands of affected patients worldwide. Patients with severe *SYNGAP1* variants frequently have very low IQ (< 50), are mostly nonverbal, and have several comorbid conditions, such as impulsivity and challenging behaviors. Damaging *SYNGAP1* variants are also causally associated with other neuropsychiatric disorders. Several groups have found damaging *SYNGAP1* variants or copy number variations in ASD patients [6–9]. Damaging de novo *SYNGAP1* mutations were also recently found in a very large cohort of schizophrenia patients [10]. Due to the range of disorders and the diversity of genetic variation associated with *SYNGAP1* pathogenicity, patients with pathogenic *SYNGAP1* variants are sometimes generally referred to as having a *SYNGAP1*-related brain disorder.

### Neurobiology of SynGAP and its importance to neurodevelopmental disorders

**Fig. 1:**
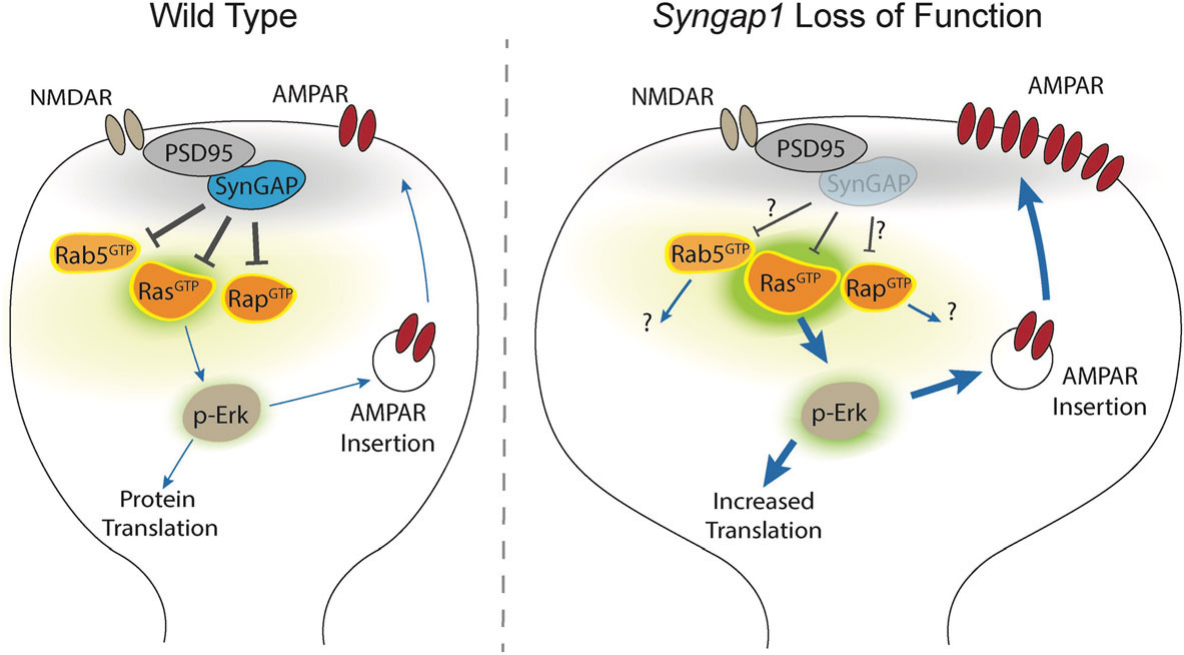
Schematic of signaling pathways regulated by *Syngap1* gene. SynGAP protein has been shown to inhibit the activation of various small GTPases. In dendritic spines, SynGAP suppresses Ras/Erk activity and limits growth-related processes including protein translation and AMPA receptor exocytosis. Reduced SynGAP protein levels causes elevated basal Ras/Erk signaling. This results in increased AMPAR surface incorporation thought to contribute to excessive excitation in neural circuits. Enhanced Ras/ERK signaling is also linked to impaired synaptic plasticity, such as altered hippocampal long-term potentiation. Although Rab5 and Rap GTPases are regulated by SynGAP, these mechanisms are poorly understood and it remains unknown how they contribute to *Syngap1*-related disorders

*SYNGAP1* encodes the synaptic Ras GTPase activating protein (SynGAP), which is highly enriched in the nervous tissue [11, 12]. RasGAPs in general regulate the dynamics of small GTPase signaling by accelerating GTP-to-GDP conversion, and thus direct inactivation, of proteins within the Ras superfamily **(**Fig. 1**)**. SynGAP has been shown to directly regulate several small GTPases, including HRAS, RAP1, RAP2, and RAB5 [11–16]. At excitatory synapses, it promotes inactivation RAS-ERK1/2 signaling, which suppresses the insertion of glutamate receptors [17, 18]. Therefore, based on the observation of elevated synaptic Ras/ERK measurements in *Syngap1* mutant mice [19], haploinsufficiency of *SYNGAP1* likely leads to increased RAS-like signaling at synapses. It is believed that aberrant GTPase dynamics in synapses contributes to alterations in cellular growth [20], synaptic plasticity [13, 19, 21], and cognitive ability [13, 22, 23]. Due to the likely direct dysregulation of RAS, MRD5 can be classified as a RASopathy, which is in the same family as neurofibromatosis type 1 or Noonan syndrome, two genetic disorders of RAS signaling that lead to cognitive impairment [24, 25].

SynGAP protein is enriched in the postsynaptic density (PSD) of neurons [11, 12]. It is known to interact with multiple proteins that are critical for normal response to circuit inputs. Among the proteins SynGAP interacts with are DLG3 and SHANK3 [26], which are themselves mutated in individuals with a breadth of neurodevelopmental disorders from ASD to ID to epilepsy [27, 28]. The PSD is enriched with proteins encoded by a high proportion of genes with pathogenic variants linked to a range neuropsychiatric disorders with cognitive impairment [29, 30]. Furthermore, SynGAP also regulates protein translation machinery [31] that modulates mGluR-dependent long-term depression in a Ras/ERK and *Fmr1*-dependent manner [32]. Altered protein translation is a major cellular process believed to contribute to ASD [33, 34]. Therefore, SynGAP regulates molecular pathways and neural substrates that are shared among distinct brain disorders. As a result, understanding the neurobiology of SynGAP is critical not only for those individuals with mutations in this gene but also more broadly for disorders with altered cognitive and social ability.

## Main text

The overarching reason for holding this inaugural meeting was to develop a consensus among the clinicians and scientists in the *SYNGAP1* community as to how best to achieve a more fundamental and profound understanding of SynGAP biology and its role in human disease. The meeting was organized as a joint effort between Bridge-the-GAP—SYNGAP Education and Research Foundation (Monica Weldon), which is a patient advocacy and awareness group focusing on *SYNGAP1*-related brain disorders, The Scripps Research Institute (Gavin Rumbaugh), and Texas Children’s Hospital (Jimmy Holder). The 2-day meeting was held at the Texas Children’s Hospital Pavilion for Women Conference Center in Houston, Texas, and all activities were designed to address four priorities/objectives.

- To bring together internationally recognized basic scientists and clinicians interested in the function of SynGAP protein, the disease substrates underlying the disorder, and the development of novel therapies for rare genetic disorders
- To further the understanding of *Syngap1* in normal brain function and to develop a consensus on the most effective avenues toward novel treatments
- To expand the *SYNGAP1* research and clinical community, including the introduction of junior scientists and clinicians, postdoctoral and clinical fellows, and graduate students to the importance of studying this and related rare diseases
- To grow the emerging international *SYNGAP1* research and clinical network to foster fully collaborative multi-laboratory basic research, to accelerate a patient registry, and to catalyze a natural history study for the advancement of patient care and treatment

These objectives were addressed through eight science-related panel sessions. In addition, there was a 2-hour luncheon where meeting attendees were encouraged to meet affected individuals and their families. There was also a banquet dinner where several caregivers shared powerful personal stories of how *SYNGAP1* has impacted their lives.

### Overview of symposium sessions

#### Session 1—Overview of family experiences with MRD5

On November 30, 2016, the meeting began with a few words from the co-chairs. They introduced the relevant stakeholders and defined the goals for the meeting. Following this, the first lecture was given by Monica Weldon, a mother of an affected child. She spoke on behalf of the patients and their families and provided an overview of the mission of Bridge-the-GAP—SYNGAP Education and Research Foundation. She also gave a general overview of the disorder from the perspective of a caregiver. Following this, a caregiver of a TSC-affected child provided their perspective in an effort to draw similarities and contrasts among caring for patients with distinct genetically defined brain disorders.

#### Session 2—Clinical aspects of MRD5

Dr. Jacques Michaud discussed his research that led to the discovery of the first patients with pathogenic *SYNGAP1* mutations [35, 36]. Dr. Michael Parker discussed findings from the Deciphering Developmental Disorders (DDD) consortium that identified *SYNGAP1* as one of the most frequent causes of classically undefined developmental delay in very young children [3–5]. Dr. Ingrid Scheffer discussed epilepsy symptomology associated with *SYNGAP1* pathogenicity, which includes frequent drop attacks and absence seizures [37].

#### Session 3—*Syngap1* neurobiology

Richard Huganir and Mary Kennedy, who were co-discovers of *Syngap1* through identification of the major protein products of the gene [11, 12], discussed molecular mechanisms of SynGAP protein function at excitatory synapses [16, 17, 38], the most well-studied cellular function of this gene. Gavin Rumbaugh provided an overview of studies in animal models that defined synaptic pathologies that occur during developmental critical periods as a consequence of *Syngap1* haploinsufficiency [19, 20, 22, 39, 40].

#### Session 4—Shared neurodevelopmental substrates in childhood brain disorders

The first day of the symposium ended with a session that highlighted the most commonly observed shared neurobiological substrates found in many genetically defined developmental brain disorders, including MRD5. Eric Klann presented his ongoing work that seeks to understand the molecular mechanisms underlying impaired mRNA translation common to genetically defined forms of ASD. Damon Page discussed his recent work in an animal model of PTEN-induced macrocephaly, which is a common genetic cause of autism. Peter Penzes presented his recent work on schizophrenia and ASD risk factors and how they influence synapse biology, circuit function, and behavior.

#### Session 5—Related genetic disorders

The first session of the second day opened with a Plenary Lecture by Huda Zoghbi. She discussed efforts in her laboratory to explore the pathogenesis of polyglutamine neurodegenerative diseases and Rett syndrome. Following the plenary, Jeffrey Noebels gave a lecture on his work investigating the cellular mechanisms affected by genes that predispose patients to epilepsy and autism and strategies for neonatal phenotypic rescue. Eric Morrow discussed the clinical and biological spectrum of Christianson syndrome, which is a rare genetic disorder caused by mutations in the solute carrier protein, NHE6. Finally, Jimmy Holder discussed his work on Phelan-McDermid Syndrome, an ID and ASD disorder believed to be caused by disruptions in the expression of the *SHANK3* gene leading to altered synapse function.

#### Session 6—Young investigator and travel awardee talks

This meeting was supported by a grant from the National Institute of Neurological Disorders and Stroke (NINDS) and the National Center for Advancing Translational Sciences (NCATS). This award enabled travel support for approximately ten postdoctoral fellows/medical residents that perform research related to *SYNGAP1/Syngap1*. Four of the awardees were selected to give short talks. Dr. Thomas Vaissiere discussed the use of monosynaptic tracing tools to understand how SynGAP promotes forebrain circuit assembly during critical periods. Dr. Maria Martin De Saavedra discussed the cellular functions of the neurodevelopmental disorder risk factor, CNTNAP2. Dr. Shu-Ling Chiu discussed the role of the ID-associated gene, *GRASP1*, in learning and synaptic function. Dr. Xiangling Meng discussed how manipulations of MeCP2 impact cellular functions related to Rett and other neurological disorders.

#### Session 7—Translational approaches to treating rare, genetically defined brain disorders

David Wylie discussed the development of two rat models of *Syngap1* pathogenicity in an effort to identify evolutionarily conserved functions of the *Syngap1* gene. Alcino Silva presented progress on identifying off-label uses for U.S. Food and Drug Administration (FDA)-approved drugs with the ability to enhance cognitive function in animal models of ID. Ben Hall of Roche Pharmaceuticals discussed industry efforts to develop therapeutics for developmental brain disorders related to MRD5. Andy Stanfield discussed efforts to improve clinical measures for intellectual disability disorders as a means to increase the likelihood of discovering effective therapeutics. Finally, Jeffrey Neul discussed the large-scale prospective natural history study of Rett Syndrome patients across the USA.

#### Session 8—What are the major neurobiological and translational barriers for developing treatments in *SYNGAP1* disorders

The final session of the meeting was an open panel discussion where participation from all stakeholders was actively encouraged. The goal of this discussion was to synthesize knowledge that emerged from the symposium in an attempt to determine the best approaches for developing effective treatments for MRD5 patients. The session had a moderator and the discussion was driven by a panel that included (1) an expert on MRD5 clinical genetics and phenotypes, (2) a representative of the pharmaceutical industry with an interest in developing treatments for genetically defined developmental brain disorders, (3) a representative from the FDA, (4) an MRD5 family member, and (5) an expert on *Syngap1* biology.

## Conclusions

From these proceedings, several areas of consensus emerged. From the clinical perspective, there was a useful discussion suggesting that the current literature describing the clinical manifestations of MRD5 likely underestimate the true prevalence of epilepsy [1–3]. It was noted that seizure types are varied across the full spectrum of known patients and some may be difficult to detect. Moreover, electroencephalogram (EEG) abnormalities appear to be universal even in patients without obvious behavioral seizures. Therefore, epilepsy co-occurrence is probably close to 100%. It was suggested that EEG is an attractive translational measure in *SYNGAP1*-related disorders due to its near-universal presence in the patient population and the observation of behavioral seizure and abnormal EEG in *Syngap1* animal models [19, 41]. The group discussion also revealed two common phenotypes that are common in MRD5 and are underreported in the current literature. Sensory processing impairments are very common in these patients. Moreover, many parents commented that their children partake in risky behaviors, such as climbing and then jumping from very high objects. These behaviors were noted as significant because they necessitate extreme vigilance from parents which leads to caregiver stress and fatigue. From the biological perspective, it was clear from the scientific panel sessions that more basic research is needed to better understand the neurobiological functions of *Syngap1*. Studies in animal models of *Syngap1* haploinsufficiency suggest that there are pleiotropic functions of this gene that may be unrelated to synaptic Ras/ERK signaling. It was mentioned that excitatory/inhibitory imbalances are observed across several research labs in animal models [19, 22, 41, 42]. There were group discussions among leaders about needing to better understand how synaptic mechanisms controlled by SynGAP contribute to overall circuit function, cognitive processing, and behavior. From the patient advocacy perspective, the meeting highlighted the need to form a robust patient registry as an initial step toward a prospective natural history study of MRD5. In response to this discussion, Bridge-the-GAP Foundation pledged to re-conceptualized their 5-year strategic plan. The new strategic plan would emphasize the primary goal of world-wide new patient engagement to grow the patient registry as quickly as possible. Post-meeting questionnaires from attendees indicated that all stakeholders were satisfied with the content and outcomes of the meeting. The questionnaires also indicated that there was a strong motivation to hold additional *SYNGAP1*-focused meetings in the future. A consensus was reached that the next meeting will focus on growing the SynGAP basic research base and to explore therapeutic strategies for treating *SYNGAP1*-related brain disorders.

## Abbreviations

ASD: Autism spectrum disorder
ID: Intellectual disability
MRD5: Autosomal mental retardation type 5
PSD: Postsynaptic density
SynGAP: Synaptic Ras GTPase activating protein
